# Clustering of characteristics associated with unplanned pregnancies: the generation R study

**DOI:** 10.1186/s12889-022-14342-y

**Published:** 2022-10-24

**Authors:** Clair A. Enthoven, Hanan El Marroun, M. Elisabeth Koopman-Verhoeff, Wilma Jansen, Mijke P. Lambregtse-van den Berg, Frouke Sondeijker, Manon H. J. Hillegers, Hilmar H. Bijma, Pauline W. Jansen

**Affiliations:** 1 Department of Child and Adolescent Psychiatry/Psychology, Erasmus University Medical Center, Rotterdam, Zuid-Holland The Netherlands; 2 Department of Psychology, Education and Child Studies, Erasmus School of Social and Behavioural Sciences, Erasmus University Rotterdam, Rotterdam, Zuid-Holland The Netherlands; 3 The Generation R Study Group, Erasmus University Medical Center, Rotterdam, Zuid-Holland The Netherlands; 4 Institute of Education and Child Studies, Leiden University, Leiden, The Netherlands; 5 Department of Social Development, Rotterdam, Zuid-Holland The Netherlands; 6 Department of Public Health, Erasmus University Medical Center, Rotterdam, Zuid-Holland The Netherlands; 7 Department of Psychiatry, Erasmus University Medical Center, Rotterdam, Zuid-Holland The Netherlands; 8 Department of Youth, parenting and education, Verwey-Jonker institute, Utrecht, The Netherlands; 9 Department of Obstetrics and Gynecology, Division of Obstetrics and Fetal Medicine, Erasmus MC Sophia, Rotterdam, the Netherlands

**Keywords:** Unplanned pregnancy, Predictors, Unwanted pregnancy, Cluster analysis, Predictors

## Abstract

**Background:**

Unplanned or unintended pregnancies form a major public health concern because they are associated with unfavorable birth outcomes as well as social adversity, stress and depression among parents-to-be. Several risk factors for unplanned pregnancies in women have previously been identified, but studies usually take a unidimensional approach by focusing on only one or few factors, disregarding the possibility that predictors might cluster. Furthermore, data on predictors in men are largely overlooked. The purpose of this study is to determine predictors of unplanned versus planned pregnancy, to determine predictors of ambivalent feelings regarding pregnancy, and to investigate how characteristics of men and women with an unplanned pregnancy cluster together.

**Methods:**

This study was embedded in Generation R, a multiethnic population-based prospective cohort from fetal life onwards. Pregnancy intention was reported by 7702 women and 5367 partners. Information on demographic, mental, physical, social, and sexual characteristics was obtained. Logistic regression, multinomial regression and cluster analyses were performed to determine characteristics that were associated with an unplanned pregnancy, with ambivalent feelings regarding the unplanned pregnancy and the co-occurrence of characteristics in women and men with unplanned pregnancy.

**Results:**

Twenty nine percent of the pregnancies were unplanned. Logistic regression analyses showed that 42 of 44 studied predictors were significantly associated with unplanned pregnancy. The most important predictors were young age, migration background, lower educational level, lower household income, financial difficulties, being single, lower cognitive ability, drug use prior to pregnancy, having multiple sexual partners in the year prior to the pregnancy, younger age of first sexual contact and a history of abortion. Multinomial regression analyses showed that a Turkish or Moroccan background, Islamic religion, little financial opportunities, being married, having ≥3 children, high educational level, more mental health and social problems and older age of first sexual contact were associated with prolonged ambivalent feelings regarding pregnancy. Different combinations of characteristics were observed in the four clusters of women and men with unplanned pregnancy.

**Conclusions:**

Many predictors are related with unplanned pregnancies, ambivalent feelings toward the pregnancy, and we identified very heterogeneous groups of women and men with unplanned pregnancies. This calls for heterogeneous measures to prevent unplanned pregnancies.

**Supplementary Information:**

The online version contains supplementary material available at 10.1186/s12889-022-14342-y.

## Background

Unplanned or unintended pregnancies are very common. More than half of the pregnancies in Latin America and the Caribbean, 45% in the United States, and 34% in Western Europe are unintended [1, 2]. In the Netherlands, about one in five women ever experiences an unplanned pregnancy, varying from 33% in women of Surinamese descent to 19% in women from Dutch descent [3]. These prevalence rates are high and indicate the common character of unintended pregnancies, but should also be interpreted keeping in mind that the terminology of planned and intended pregnancy varies and are often used interchangeably [4]. Moreover, both unintended and unplanned pregnancies can be wanted (but perhaps mistimed), unwanted or ambivalent. In the current study, unplanned pregnancy (UP) was examined; women were asked whether the pregnancy was planned or not, and how they felt about the pregnancy in case this was unplanned.

Unplanned or unintended pregnancies are associated with unfavorable birth outcomes, including preterm birth and low birthweight (< 2500 g) [5, 6] and may be associated with pregnancy loss and neonatal mortality [7]. Women with unplanned pregnancies lack preconception care and embryonic and fetal development are more frequently exposed to illicit drugs use, alcohol and smoking, as well as poor nutrition, with long-lasting impact on health and development [6, 8–11]. Also, late booking for antenatal care may result in lack of obstetric care and screening and in some cases unattended birth. Furthermore, unplanned pregnancy is associated with higher risk of gestational diabetes, more vaginal bleeding during child-bearing, social adversity, stress and depression among future parents [12–16]. Lastly, implications are long-lasting with lower self-esteem, mental health problems, and suboptimal cognitive development being more frequent in children born from unplanned pregnancy [7, 17–19]. However, development of both public health approaches and care programs has been impeded by lack of understanding which combination of risk factors is associated with unplanned pregnancy.

Several risk factors for unplanned pregnancies have been identified for women. Firstly, younger age (< 20 years) or older age (> 35 years), lower educational level and being single have been reported as risk factors [20, 21]. Secondly, a history of unplanned pregnancies, abortions and treatment for a sexually transmitted disease (STD) have been associated with unplanned pregnancies [22]. Thirdly, mental health problems, illicit drugs use and binge drinking have been associated with unplanned pregnancy [21, 23, 24]. Finally, sexual violence, intimate partner violence and coercion are associated with unplanned pregnancy [25]. Five to 7 % of the women who experienced rape reported that they got pregnant as a result of the rape [26, 27]. Unfortunately, to date, most studies lack a multi-dimensional approach in evaluating only one or a few factors, resulting in limited insight of the context and patterns that may be involved. Also, factors related to men are largely ignored. Only few studies reported lifetime sexual partners, having financial difficulties, history of sexual abuse and rural residence as potential male risk factors for unplanned pregnancy [28, 29]. This results in a limited understanding of the context in which unplanned pregnancy occurs, with disproportionate emphasis on the role of women in unplanned pregnancy.

The aims of this exploratory study are firstly to determine predictors of unplanned pregnancy versus planned pregnancy in a large population-based urban birth cohort in Rotterdam, the Netherlands. Secondly, to determine predictors of ambivalent feelings toward the pregnancy in women with unplanned pregnancy. We will thirdly investigate the individual and contextual characteristics (demographic, mental and physical health, social, sexual behavior and substance use) of women and men with unplanned pregnancy and how these characteristics cluster together [30]. This knowledge will provide insight in possible targets for prevention of unplanned pregnancies or interventions aimed at minimizing unfavorable outcomes in unplanned pregnancies.

## Methods

### Study population

This study was embedded in Generation R, a multiethnic population-based prospective cohort from fetal life onwards, designed to identify early environmental and genetic determinants of growth, development, and health. The cohort has previously been described in detail [31, 32]. Briefly, all pregnant women who resided in Rotterdam at the time of their delivery and with a date of delivery between April 2002 and January 2006 were invited to participate. Enrollment was aimed at gestational age < 18 weeks, but was possible until birth of the child. In total, 9778 (response rate 61%) women and 6347 partners were enrolled in Generation R. In case women participated in the study with multiple pregnancies (*n* = 642 women), only the first pregnancy (of which 19.5% was unplanned) was included in the current study (*n* = 625 excluded second or third pregnancies of which 25.1% was unplanned). Women with missing information on pregnancy planning were excluded (*n* = 1451), most of these (73%) did not fill out the questionnaire. The final study sample consisted of 7702 women and 5367 partners (Fig. S1). The Medical Ethics Committee of Erasmus MC in Rotterdam, the Netherlands, has approved the study in accordance with the Declaration of Helsinki of the World Medical Association. Written informed consent was obtained from all participants.

### Measurements

#### Main outcome

Pregnancy planning was measured using a self-report questionnaire at first research visit. Women reported whether their pregnancy was planned or not. Pregnancy planning was compared with pre-pregnancy folic acid use and contraceptive use at conception. In sensitivity analyses, we excluded those who reported both an unplanned pregnancy and pre-pregnancy folic acid intake (*n* = 93) and those who reported a planned pregnancy and contraceptive use at conception (*n* = 167). For contraceptive use, women were asked until which month and year they used contraceptives. Contraceptive use after conception was defined as ‘yes’ when the contraceptives were taken in the month after the date of the last menstruation.

In case of an unplanned pregnancy, women reported how they felt about the pregnancy using the following four answering categories: “pleased from the start” (*n* = 1157); “initially mixed feelings” (*n* = 827); “still mixed feelings” (*n* = 90); or “mostly unhappy about the pregnancy” (*n* = 18). Few women reported having “still mixed feelings” or “mostly unhappy”, and thus we categorized the feelings towards the unplanned pregnancies into: wanted from the start (*n* = 1157), initially ambivalent feelings (*n* = 827) and prolonged ambivalent feelings (*n* = 108). Women who entered the study at birth did not receive the question about the feelings towards the pregnancy resulting in missing information for 168 women.

#### Demographic characteristics

A wide range of characteristics was examined as potential predictors of unplanned pregnancies. Detailed information is listed in Table S1. For both women and men, age at conception was calculated by subtracting the gestational age at birth from parental age at birth. Ethnic background for women and men was determined by the country of birth of their parents. For women, ethnic background, educational level and marital status was measured by questionnaire in the 12th week of gestation. Religion, whether or not having a paid job, net household income and financial difficulties were measured using a questionnaire in the 30th week of gestation. Parity was obtained from medical records or obtained by questionnaire if medical records were not available. For men, ethnic background, educational level, whether or not having a paid job were measured by questionnaire in the second trimester of pregnancy.

#### Mental health characteristics

For women, adverse experiences in childhood were measured using the Childhood Trauma Questionnaire (CTQ), perceived parental rearing was measured using the ‘Own memories on parenting questionnaire’ (s-EMBU), history of depression, anxiety and eating disorders were measured using vignettes in the 20th week of gestation [33, 34]. Self-esteem was measured using the Rosenberg Self-Esteem Scale in week 30 of gestation [35]. Maternal cognitive ability was measured using the short version of the Raven’s Progressive Matrices at the research center when the child was 5 years old [36]. We expect that this measure is relatively stable, and not influenced by whether or not the pregnancy was planned. For men, history of depression and anxiety were measured using vignettes at 18–25 weeks of gestation.

The Composite International Diagnostic Interview (CIDI) was obtained among a subgroup of women (*n* = 928) and men (*n* = 827). Sensitivity and specificity of the vignettes were calculated with the CIDI as gold standard. This resulted in a good sensitivity and specificity for depression and eating disorder, and fair sensitivity and good specificity for anxiety in both women and men (Table S1).

#### Physical health characteristics

Chronic somatic diseases were measured using a questionnaire at 12 weeks of gestation for women and 18–25 weeks for men. Pre-pregnancy body mass index (BMI) was calculated based on height (m) and weight (kg) as self-reported by women in a questionnaire at enrolment. For men, weight and height was measured at the research center in gestational age < 18 weeks.

#### Social characteristics

For women, relational difficulties were measured using the Dutch Long-Lasting Difficulties (LLD) list and number of good friends was determined using a questionnaire in week 30 of gestation [37]. For both women and men, history of delinquency was self-reported in week 20 of gestation [38].

#### Substance use

Pre-pregnancy alcohol use, cigarette smoking and drug use were measured using a questionnaire in week 12 of gestation. Women reported about themselves and their partners. Men also reported about their own substance use, but women’s reports were preferred because of less missing data. The agreement between women’s and men’s reports was very high (86–96%).

#### Sexual behavior

The number of sexual partners in the year prior to pregnancy and history of treatment for a STD were reported by the women about themselves and about their partners. Age of first sexual contact and history of abortion were self-reported by women only using a questionnaire in the 12th week of gestation.

### Statistical analyses

Descriptive information of the study population was presented separately for women and men with a planned pregnancy versus unplanned pregnancy. Then, non-response analyses in men were performed based on available information reported by the women, using chi-square tests. To avoid complete-case bias due to missing information on the predictors, multiple imputation procedures were performed using Multivariate Imputations by Chained Equations (MICE) with 50 imputed datasets and 100 iterations [39]. For the first aim, univariate logistic regression models were performed on the imputed data, with pregnancy planning as outcome variable (reference: planned pregnancy) in both women and men. Pooled crude Odds Ratios (ORs) with 95% Confidence Intervals (CI) were presented. Sensitivity analyses were performed excluding those who reported both an unplanned pregnancy and pre-pregnancy folic acid intake (*n* = 93) and excluding those who reported a planned pregnancy and contraceptive use at conception (*n* = 167). For the second aim, univariate multinomial logistic regression models were performed with feelings towards the pregnancy as outcome variable (reference: wanted from the start) on the imputed data for women with an unplanned pregnancy only.

For the third aim, hierarchical cluster analyses were performed among men and women with unplanned pregnancy to identify clusters of characteristics. Cluster analysis aims to group data with close proximity to each other together in a cluster [30]. All significant predictors from the logistic regression models of the first aim were included in the cluster analyses for women and men separately. Age at conception, BMI and age of first sexual contact were continuously included in the cluster analyses to increase precision. The agglomerative hierarchical algorithm was used with Gower’s distance as dissimilarity matrix because of the mixed type of data [40, 41]. The non-imputed dataset was used for cluster analysis, because the calculation of Gower’s dissimilarity allows for missing values. However, for 148 women and 302 men the number of missing data was too high, resulting in missing values in the dissimilarity matrix, hence they were excluded. The daisy function from the R package ‘cluster’ was used to calculate Gower’s dissimilarity matrix [42]. Agglomerative hierarchical cluster analyses were performed using the agnes function from the R package ‘cluster’ [42]. Different linkage methods in the agnes function were compared and Ward’s method was chosen because it had the highest agglomerative coefficient (0.99 in women and men). The agglomerative coefficient describes the strength of the clustering structure, the closer to 1 suggests a more balanced clustering structure. Cluster validation and the number of clusters was chosen based on several validation parameters from the cluster.stats function from the R package ‘fpc’ [43]. The cluster validation parameters were the largest average distance between clusters, the smallest average distance within clusters, the highest average Silhouette width, the highest Dunn index, and the highest separation index.

For visualization of the clusters, a heat map was created using the ‘pheatmap’ R package [44]. We used IBM SPSS Statistics 25, R 3.6.1 and R Studio 1.1.456 for data preparation and analyses.

## Results

### General characteristics

The women were on average 29.5 ± 5.3 years old and the men 32.5 ± 5.7 years at conception. Around half of the women (49.8%) and less than half of the men (37.7%) had a migration background, most had a Surinamese (8.9 and 6.9%, respectively), Turkish (8.8 and 6.7%) and non-Dutch European (8.1 and 6.0%) background. Most women were married (48.5%) or cohabiting (36.8%), and some were single (14.7%). Most women were nulliparous (60.9%), 27.3% had already a child, 9.2% had two children and 2.6% had three or more children. In total, 29.3% of the women had an unplanned pregnancy, of whom 55.3% were immediately happy about it, 39.5% had initially ambivalent feelings and 5.3% had prolonged ambivalent feelings. All participant characteristics are shown in Table 1, separately for planned and unplanned pregnancies.

**Table 1:** Frequencies and univariate logistic regression analyses describing associations between predictors and pregnancy planning for women and men separately

	Women (***N*** = 7702)			Men (***N*** = 5367)		
	Planned (***N*** = 5442)	Unplanned (***N*** = 2260)	OR (95% CI) for Unplanned pregnancy	Planned (***N*** = 4087)	Unplanned (***N*** = 1280)	OR (95% CI) for Unplanned pregnancy
***Demographic information***						
**Age**						
< 20 years	105 (2.0%)	250 (11.3%)	9.15 (7.16–11.69)	13 (0.4%)	46 (4.4%)	13.04 (7.10–23.97)
20–25 years	705 (13.1%)	582 (26.2%)	3.20 (2.78–3.70)	168 (4.6%)	181 (17.3%)	4.98 (3.93–6.33)
25–30 years	1555 (29.0%)	550 (24.8%)	1.38 (1.21–1.58)	797 (21.8%)	275 (26.3%)	1.81 (1.50–2.18)
30–35 years	2211 (41.2%)	569 (25.6%)	1.00	1562 (42.6%)	282 (26.9%)	1.00
> 35 years	794 (14.8%)	269 (12.1%)	1.31 (1.11–1.54)	1123 (30.7%)	263 (25.1%)	1.26 (1.04–1.51)
N missing	72	40	NA	424	233	NA
**Migration background**						
Dutch	3094 (57.4%)	735 (33.0%)	1.00	2611 (67.5%)	507 (44.9%)	1.00
Indonesian	170 (3.2%)	65 (2.9%)	1.60 (1.19–2.16)	129 (3.3%)	45 (4.0%)	1.80 (1.27–2.57)
Cape Verdian	122 (2.3%)	181 (8.1%)	6.16 (4.83–7.86)	72 (1.9%)	57 (5.0%)	4.43 (3.14–6.26)
Moroccan	342 (6.3%)	145 (6.5%)	1.78 (1.45–2.20)	154 (4.0%)	57 (5.0%)	1.88 (1.38–2.56)
Dutch Antilles	96 (1.8%)	170 (7.6%)	7.36 (5.67–9.57)	72 (1.9%)	71 (6.3%)	5.03 (3.61–7.00)
Surinamese	343 (6.4%)	335 (15.0%)	4.09 (3.45–4.85)	212 (5.5%)	134 (11.9%)	3.24 (2.57–4.08)
Turkish	440 (8.2%)	228 (10.2%)	2.17 (1.81–2.59)	230 (5.9%)	103 (9.1%)	2.43 (1.92–3.07)
European	470 (8.7%)	149 (6.7%)	1.33 (1.09–1.62)	233 (6.0%)	69 (6.1%)	1.55 (1.17–2.05)
Asian	154 (2.9%)	76 (3.4%)	2.08 (1.56–2.77)	77 (2.0%)	40 (3.5%)	2.59 (1.76–3.83)
Other	163 (3.0%)	143 (6.4%)	3.66 (2.89–4.65)	78 (2.0%)	47 (4.2%)	3.14 (2.17–4.55)
N missing	48	33	NA	219	150	NA
**Educational level**						
Low	1159 (22.0%)	841 (39.3%)	4.49 (3.81–5.30)	709 (20.3%)	331 (34.0%)	3.34 (2.75–4.07)
Mid-low	1546 (29.4%)	781 (36.5%)	3.13 (2.66–3.69)	894 (25.6%)	326 (33.5%)	2.50 (2.05–3.04)
Mid-high	1125 (21.4%)	293 (13.7%)	1.63 (1.35–1.97)	706 (20.2%)	146 (15.0%)	1.46 (1.15–1.85)
High	1436 (27.3%)	227 (10.6%)	1.00	1189 (34.0%)	171 (17.6%)	1.00
N missing	176	118	NA	589	306	NA
**Paid job; no**	1004 (21.0%)	662 (44.2%)	3.02 (2.70–3.38)	219 (6.6%)	130 (14.5%)	2.46 (1.99–3.05)
N missing	1131	763	NA	778	386	NA
**Household income**						
< €1200/month	513 (12.5%)	590 (40.4%)	6.05 (5.30–6.91)			
€1200–€2000/month	703 (17.1%)	355 (24.3%)	2.73 (2.35–3.19)			
> €2000/month	2892 (70.4%)	516 (35.3%)	1.00			
N missing	1334	799	NA			
**Financial difficulties**						
No	3457 (84.2%)	914 (63.2%)	1.00			
Some	547 (13.3%)	416 (28.7%)	2.75 (2.40–3.16)			
Great	100 (2.4%)	117 (8.1%)	4.16 (3.28–5.28)			
N missing	1338	813	NA			
**Religion**						
Not religious	2869 (63.7%)	891 (56.2%)	1.00			
Christian	843 (18.7%)	333 (21.0%)	1.29 (1.13–1.48)			
Hindustan	90 (2.0%)	45 (2.8%)	1.51 (1.07–2.11)			
Islamic	596 (13.2%)	262 (16.5%)	1.36 (1.19–1.57)			
Other religion	104 (2.3%)	55 (3.5%)	1.73 (1.24–2.41)			
N missing	940	674	NA			
**Marital status**						
Married	2927 (54.9%)	723 (33.1%)	1.00			
Cohabiting	2058 (38.6%)	711 (32.5%)	1.41 (1.25–1.59)			
Single	350 (6.6%)	753 (34.4%)	8.61 (7.42–10.01)			
N missing	107	73	NA			
**Parity**						
0	3255 (61.3%)	1310 (60.1%)	1.21 (1.07–1.36)			
1	1543 (29.0%)	502 (23.0%)	1.00			
2	413 (7.8%)	274 (12.6%)	2.06 (1.71–2.47)			
≥ 3	102 (1.9%)	92 (4.2%)	2.98 (2.22–3.99)			
N missing	129	82	NA			
***Mental health***						
**Childhood trauma; score (SD)**	−0.10 (0.90)	0.28 (1.19)	1.38 (1.31–1.47)			
N missing	1383	781	NA			
**Perceived parental rearing**						
Emotional warmth; score (SD)	0.03 (1.00)	−0.08 (1.00)	0.84 (0.74–0.97)			
N missing	2734	1277	NA			
Overprotection; score (SD)	−0.04 (0.98)	0.13 (1.05)	1.19 (1.11–1.26)			
N missing	1489	849	NA			
Rejection; score (SD)	−0.08 (0.91)	0.22 (1.20)	1.26 (1.19–1.34)			
N missing	1525	884	NA			
**History of depression; yes**	1059 (25.7%)	564 (36.5%)	1.73 (1.45–2.07)	524 (15.5%)	178 (19.0%)	1.40 (1.12–1.75)
N missing	1316	714	NA	702	341	NA
**History of anxiety; yes**	598 (14.4%)	308 (19.6%)	1.53 (1.27–1.84)	275 (8.1%)	93 (9.8%)	1.34 (1.02–1.77)
N missing	1284	686	NA	692	333	NA
**History of eating disorder; yes**	364 (8.9%)	175 (11.5%)	1.42 (1.14–1.76)			
N missing	1347	742	NA			
**Self-esteem; score (SD)**	0.08 (0.94)	−0.24 (1.12)	0.73 (0.68–0.77)	−0.08 (0.91)	0.30 (1.22)	
N missing	1526	877	NA	667	325	
**Cognition (IQ)**						
< 70	151 (4.0%)	95 (7.1%)	2.16 (1.69–2.76)			
70–85	659 (17.4%)	381 (28.6%)	1.91 (1.68–2.17)			
≥ 85	2974 (78.6%)	858 (64.3%)	1.00			
N missing	1658	926	NA			
***Physical health***						
**Chronic somatic disease; yes**	397 (7.6%)	155 (7.1%)	0.94 (0.77–1.14)	269 (7.7%)	76 (7.8%)	1.09 (0.83–1.42)
N missing	188	79	NA	615	303	NA
**BMI prior to pregnancy**						
< 20	684 (14.7%)	381 (19.5%)	1.39 (1.20–1.61)	151 (3.7%)	66 (5.2%)	1.55 (1.14–2.10)
20–25	2672 (57.5%)	1011 (51.7%)	1.00	1896 (46.5%)	607 (47.9%)	1.14 (0.99–1.30)
25–30	907 (19.5%)	354 (18.1%)	1.04 (0.90–1.20)	1687 (41.4%)	477 (37.7%)	1.00
≥ 30	383 (8.2%)	208 (10.6%)	1.41 (1.17–1.70)	343 (8.4%)	116 (9.2%)	1.20 (0.95–1.51)
N missing	796	306	NA	10	14	NA
***Social factors***						
**Relational difficulties; score (SD)**	−0.15 (0.87)	0.39 (1.21)	1.62 (1.53–1.71)	−0.08 (0.91)	0.30 (1.22)	1.41 (1.32–1.50)
N missing	892	572	NA	667	325	NA
**History of delinquency**						
No crimes	2554 (61.1%)	796 (50.7%)	1.00	1266 (40.6%)	309 (35.4%)	1.00
Petty crimes	702 (16.8%)	321 (20.4%)	1.36 (1.17–1.58)	355 (11.4%)	98 (11.2%)	1.10 (0.86–1.42)
More serious crimes	927 (22.2%)	454 (28.9%)	1.50 (1.31–1.70)	1497 (48.0%)	467 (53.4%)	1.28 (1.10–1.50)
N missing	1259	689	NA	969	406	NA
**≤ 1 close friend; yes**	151 (5.0%)	107 (8.6%)	1.90 (1.48–2.44)			
N missing	2401	1018	NA			
***Substance use***						
**Alcohol intake prior to pregnancy**						
< 1 glass/week	2950 (62.6%)	1078 (53.5%)	1.00	662 (20.9%)	252 (28.4%)	1.00
1–6 glasses/week	613 (13.0%)	277 (13.7%)	0.77 (0.68–0.87)	1702 (53.8%)	415 (46.7%)	0.68 (0.57–0.80)
≥ 1 glass/day	1148 (24.4%)	661 (32.8%)	0.76 (0.62–0.94)	798 (25.2%)	221 (24.9%)	0.70 (0.58–0.85)
N missing	2157	1025	NA	925	392	NA
**Smoking prior to pregnancy**						
No	2950 (62.6%)	1078 (53.5%)	1.00	2274 (58.3%)	577 (47.5%)	1.00
< 5 cigarettes/day	613 (13.0%)	277 (13.7%)	1.21 (1.03–1.41)	614 (15.8%)	191 (15.7%)	1.23 (1.02–1.48)
≥ 5 cigarettes/day	1148 (24.4%)	661 (32.8%)	1.53 (1.36–1.72)	1010 (25.9%)	447 (36.8%)	1.74 (1.51–2.01)
N missing	731	244	NA	189	65	NA
**Drug use prior to pregnancy; yes**	297 (5.7%)	278 (12.8%)	2.42 (2.04–2.87)	347 (8.9%)	232 (19.2%)	2.40 (2.00–2.87)
N missing	211	88	NA	170	70	NA
***Sexual behavior***						
**> 1 sexual partner in the year prior to pregnancy; yes**	265 (5.5%)	276 (14.0%)	2.62 (2.16–3.17)	173 (4.5%)	156 (13.9%)	3.38 (2.70–4.24)
N missing	656	285	NA	256	157	NA
**History of treatment for STD; yes**	485 (10.2%)	303 (15.4%)	1.63 (1.38–1.92)	235 (6.2%)	108 (9.5%)	1.63 (1.28–2.07)
N missing	674	297	NA	318	147	NA
**Age of first sexual contact**						
< 16 years	575 (12.7%)	395 (21.7%)	2.23 (1.86–2.67)			
16–20 years	3066 (67.7%)	1156 (63.4%)	1.24 (1.07–1.44)			
≥ 21 years	885 (19.6%)	271 (14.9%)	1.00			
N missing	916	438	NA			
**History of abortion; yes**	565 (19.8%)	464 (35.7%)	2.21 (1.91–2.56)			
N missing	2589	962	NA			

Values indicate odds ratios (OR) and 95% confidence intervals (CI) with planned pregnancy as reference

*UP* Unplanned pregnancy

### Non-response analysis

The partners participated in the Generation R study in 75.1% of the planned pregnancies and 56.6% of the unplanned pregnancies. Partners who did not participate more often had a migration background, and were more often < 25 years or ≥ 35 years old. They were less likely to use alcohol, but more likely to smoke or use drugs prior to pregnancy. These non-participating partners more often had had multiple sexual partners in the year prior to pregnancy than partners who did participate, as reported by the women. No difference was found regarding a history of STD (treatment).

### Predictors for unplanned pregnancy

Almost all variables were significantly associated with pregnancy planning (Table 1). For example, women aged < 20 years (OR = 9.15, 95% CI = 7.16–11.69), 20–25 years (OR = 3.20, 95% CI = 2.78–3.70), 25–30 years (OR = 1.38, 95% CI = 1.21–1.58) and ≥ 35 years (OR = 1.31, 95% CI = 1.11–1.54) more often had an unplanned pregnancy as compared to women aged 30–35 years (reference group). Next to age, the factors most strongly associated with unplanned pregnancies in women were a Dutch Antillean (OR = 7.36, 95% CI = 5.67–9.57), Cape Verdean (OR = 6.16, 95% CI = 4.83–7.86) and Surinamese (OR = 4.09, 95% CI = 3.45–4.85) background, lower educational level (OR = 4.49 95% CI = 3.81–5.30), lower household income (OR = 6.05, 95% CI = 5.30–6.91), financial difficulties (OR = 4.16, 95% CI = 3.28–5.28), being single (OR = 8.61, 95% CI = 7.42–10.01), lower cognitive ability (OR = 2.16, 95% CI = 1.69–2.76), drug use prior to pregnancy (OR = 2.42, 95% CI = 2.04–2.87), having multiple sexual partners in the year prior to the pregnancy (OR = 2.62, 95% CI = 2.16–3.17), age of first sexual contact < 16 years (OR = 2.23, 95% CI = 1.86–2.67), and a history of previous induced abortion (OR = 2.21, 95% CI = 1.91–2.56). In men, the most prominent associations were found for age < 20 years (OR = 13.04, 95% CI = 7.10–23.97), Dutch Antillean (OR = 5.03, 95% CI = 3.61–7.00), Cape Verdean (OR = 4.43, 95% CI = 3.14–6.26) and Surinamese (OR = 3.24, 95% CI = 2.57–4.08) background, lower educational level (OR = 3.34, 95% CI = 2.75–4.07), drug use prior to pregnancy (OR = 2.40, 95% CI = 2.00–2.87), and multiple sexual partners in the year prior to the pregnancy (OR = 3.38, 95% CI = 2.70–4.24). In contrast, only a chronic somatic disease was not associated with pregnancy planning in either women or men. Sensitivity analyses excluding those who reported an unplanned pregnancy combined with prenatal folic acid use and those who reported a planned pregnancy while still using contraceptives during the period of conception showed similar results (data not shown).

### Predictors for ambivalent feelings towards the unplanned pregnancy

Table 2 shows which predictors were associated with initially or prolonged ambivalent feelings towards the unplanned pregnancy as compared to wanted from the start (*N* = 1157; reference group). For example, women aged < 20 years (OR = 1.60, 95% CI = 1.24–2.06), 20–25 years (OR = 1.48, 95% CI = 1.17–1.87) and ≥ 35 years (OR = 1.21, 95% CI = 1.01–1.46) had a significantly more often initially ambivalent feelings as compared to women aged 30–35 years (reference group). Women aged ≥35 years also had significantly more often prolonged ambivalent feelings as compared to women aged 30–35 years (OR = 1.91, 95% CI = 1.27–2.86). Predictors associated with only initially but not prolonged ambivalent feelings were Cape Verdean background, low and mid-low educational levels, being single, having 2 children, IQ score < 70, BMI of 25–30, smoking < 5 cigarettes/day prior to pregnancy and drug use prior to pregnancy. Predictors associated with both initially and prolonged ambivalent feelings were having a Turkish background, Islamic religion, lower household income, more financial difficulties, having ≥3 children, higher childhood trauma score, having a history of depression or anxiety, lower self-esteem score, higher relational difficulties score. Predictors associated with prolonged ambivalent feelings only were having a Moroccan background, high educational level, not having a paid job, being married, and age of first sexual contact ≥21 years.

**Table 2:** Univariate multinomial logistic regression analyses describing associations between predictors and feelings about the pregnancy among women with an unplanned pregnancy

	Initially ambivalent feelings OR (95% CI) (***N*** = 827)	Prolonged ambivalent feelings OR (95% CI) (***N*** = 108)
***Demographic predictors***		
**Age**		
< 20 years	1.60 (1.24–2.06)	0.93 (0.53–1.64)
20–25 years	1.48 (1.17–1.87)	0.87 (0.52–1.46)
25–30 years	1.19 (0.98–1.46)	1.39 (0.87–2.23)
30–35 years	1.00	1.00
≥ 35 years	1.21 (1.01–1.46)	1.91 (1.27–2.86)
**Ethnic background**		
Dutch	1.00	1.00
Indonesian	0.89 (0.49–1.60)	0.82 (0.19–3.60)
Cape Verdean	2.38 (1.68–3.38)	1.94 (0.87–4.30)
Moroccan	1.40 (0.95–2.07)	2.37 (1.12–4.98)
Dutch Antilles	1.24 (0.87–1.77)	0.97 (0.39–2.42)
Surinamese	1.49 (1.13–1.97)	0.98 (0.47–2.02)
Turkish	1.90 (1.37–2.64)	4.13 (2.29–7.45)
European	1.14 (0.77–1.67)	0.76 (0.26–2.23)
Asian	1.17 (0.68–1.99)	2.49 (0.97–6.39)
Other	1.26 (0.85–1.87)	1.28 (0.51–3.20)
**Religion**		
No religion	1.00	1.00
Christian	1.13 (0.87–1.46)	0.65 (0.30–1.37)
Hindustan	1.11 (0.64–1.92)	0.75 (0.15–3.79)
Islamic	1.34 (1.05–1.72)	2.38 (1.49–3.80)
Other	1.06 (0.59–1.90)	1.10 (0.33–3.66)
**Educational level**		
Low	1.83 (1.44–2.32)	0.71 (0.47–1.10)
Mid-low	1.46 (1.15–1.83)	0.56 (0.36–0.88)
Mid-high	1.02 (0.81–1.28)	0.54 (0.34–0.87)
High	1.00	1.00
**Having no paid job**	1.44 (0.18–1.76)	1.75 (1.11–2.75)
**Household income**		
< €1200/month	2.22 (1.87–2.63)	2.43 (1.62–3.64)
€1200–2000/month	1.69 (1.37–2.08)	1.97 (1.27–3.07)
≥ €2000/month	1.00	1.00
**Financial difficulties**		
No	1.00	1.00
Some	1.59 (1.26–2.01)	1.89 (1.16–3.06)
Great	1.74 (1.20–2.51)	2.42 (1.20–4.88)
**Marital status**		
Married	1.00	1.00
Cohabiting	0.80 (0.64–1.01)	0.40 (0.23–0.70)
Single	1.48 (1.19–1.85)	1.07 (0.68–1.67)
**Parity**		
0	1.19 (0.95–1.50)	0.58 (0.36–0.92)
1	1.00	1.00
2	2.19 (1.65–2.90)	1.38 (0.77–2.50)
≥ 3	2.23 (1.39–3.57)	2.54 (1.18–5.47)
***Mental health***		
**Childhood trauma score**	1.13 (1.04–1.23)	1.29 (1.11–1.50)
**Perceived parental rearing**		
Emotional warmth score	0.90 (0.79–1.03)	0.66 (0.38–1.13)
Overprotection score	1.08 (0.99–1.19)	1.20 (0.97–1.50)
Rejection score	1.09 (1.01–1.18)	1.17 (1.00–1.38)
**History of depression, yes**	1.35 (1.08–1.67)	2.27 (1.44–3.57)
**History of anxiety, yes**	1.54 (1.20–1.97)	2.07 (1.22–3.51)
**History of eating disorder, yes**	1.23 (0.89–1.69)	1.74 (0.94–3.22)
**Self-esteem score**	0.82 (0.75–0.91)	0.62 (0.52–0.75)
**Cognition, IQ score**		
< 70	1.64 (1.20–2.25)	1.55 (0.76–3.18)
70–85	1.21 (0.95–1.54)	1.34 (0.85–2.10)
≥ 85	1.00	1.00
***Physical health***		
**Chronic somatic disease**	1.14 (0.79–1.64)	1.04 (0.47–2.33)
**BMI prior to pregnancy**		
< 20	1.08 (0.85–1.38)	1.23 (0.68–2.23)
20–25	1.00	1.00
25–30	1.29 (1.04–1.62)	1.60 (0.96–2.67)
≥ 30	1.02 (0.84–1.24)	0.77 (0.48–1.24)
***Social predictors***		
**Relational difficulties, score**	1.24 (1.14–1.35)	1.48 (1.27–1.72)
**History of delinquency**		
No crimes	1.00	1.00
Petty crimes	0.89 (0.73–1.08)	0.79 (0.50–1.24)
Serious crimes	1.20 (0.97–1.49)	1.04 (0.65–1.67)
**≤1 Close friend, yes**	0.94 (0.63–1.40)	1.40 (0.68–2.88)
***Substance use***		
**Alcohol use prior to pregnancy**		
< 1 glass/week	1.00	1.00
1–6 glasses/week	0.82 (0.62–1.08)	0.86 (0.47–1.55)
≥ 1 glass/day	1.03 (0.80–1.33)	1.20 (0.71–2.03)
**Smoking prior to pregnancy**		
No	1.00	1.00
< 5 cigarettes/day	0.83 (0.71–0.97)	1.18 (0.85–1.65)
≥ 5 cigarettes/day	0.99 (0.80–1.24)	0.82 (0.53–1.27)
**Drug use prior to pregnancy, yes**	1.56 (1.11–1.91)	1.17 (0.63–2.14)
***Sexual risk behavior***		
**> 1 sexual partner**	1.26 (0.97–1.64)	1.33 (0.76–2.32)
**History of treatment for STD**	1.27 (0.99–1.62)	0.78 (0.41–1.48)
**Age of first time sexual contact**		
< 16 years	0.97 (0.77–1.22)	0.34 (0.19–0.61)
16–20 years	0.91 (0.76–1.09)	0.64 (0.43–0.95)
≥ 21 years	1.00	1.00
**History of induced abortion**	1.07 (0.86–1.34)	1.05 (0.68–1.64)

Values indicate odds ratios and 95% confidence intervals. Reference group: “Wanted from the start”

### Clusters of unplanned pregnancy in women

**Fig. 1:**
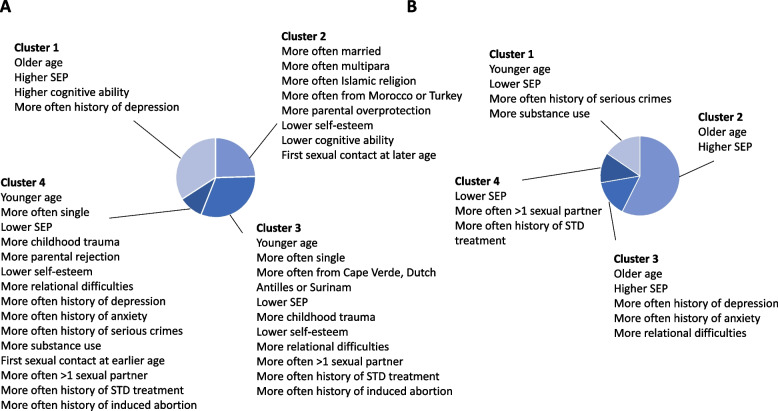
**A** + **B**: Pie diagrams showing a brief overview of the most discriminative predictors for all four clusters in women and men separately. A detailed overview is shown in Fig. S2 (women), Fig. S3 (men) and Table S3 (women) and Table S5 (men). SEP: socioeconomic position; STD: sexually transmitted disease

The cluster analyses identified four clusters of women with an unplanned pregnancy; these were selected based on cluster validation parameters and visual inspection of the heat map (Table S2 and Fig. S2A). The first cluster (*n* = 721) consisted of women with a relatively older age, and a higher educational level, income and cognitive ability. They were most often cohabiting and relatively often had a history of depression. The second cluster (*n* = 518) consisted mainly of married multipara women with a migration background, often from Morocco and Turkey, and were often Islamic. They perceived more overprotection by their parents in their childhood, had a lower self-esteem, lower cognitive ability and had their first sexual contact at a relatively older age. The third cluster (*n* = 665) consisted of relatively young, mainly single women with migration background, often from Cape Verde, Dutch Antilles and Surinam. They had a relatively low educational level, income and cognitive ability, and more often had financial difficulties. They had on average higher childhood trauma scores, and perceived their parents’ rearing as less warm with relatively high levels of overprotection and rejection. They had a lower self-esteem, and a higher relational difficulties score. They often had > 1 sexual partner in the year prior to pregnancy, a history of STD treatment and induced abortion. The fourth cluster (*n* = 208) consisted also of relatively young, mainly single women with a low educational level and income and who had more financial difficulties. In contrast with cluster 3, the women from cluster 4 were more often Dutch, and reported more childhood trauma and more parental rejection. They more often had a history of depression and anxiety, a lower self-esteem and higher relational difficulties score. Most of them had a history of petty or serious crimes, were smoking ≥5 cigarettes/day prior to pregnancy and almost all used drugs (occasionally) prior to pregnancy. They were relatively young when they had their first sexual contact, and more often had > 1 sexual partner in the year prior to pregnancy, a history of STD treatment and induced abortion. A brief summary of the clusters is visualized in Fig. 1A. More details on the clusters can be found in Table S3 and a detailed visual overview can be found in Fig. S3.

### Clusters of unplanned pregnancy in men

We also identified four clusters of men with an unplanned pregnancy; these were selected identically to the women based on cluster validation parameters and visual inspection of the heat map (Table S4 and Fig. S2B). The first cluster (*n* = 168) consisted of relatively young men with a lower educational level, more often having no paid job, and often a history of serious crimes. They were more often smoking ≥5 cigarettes/day prior to pregnancy and almost all occasionally used drugs prior to pregnancy. The second cluster (*n* = 623) consisted of relatively older men with a higher educational level. They had no remarkable high values regarding mental health, substance use and sexual behavior. The third cluster (*n* = 162) also consisted of relatively older men with a higher educational level. In contrast to cluster two, they very often had a history of depression and/or anxiety and a higher score on relational difficulties. The fourth cluster (*n* = 133) consisted of men with a relatively lower educational level. They very often had multiple sexual partners in the year prior to pregnancy and a history of STD treatment. A brief summary of the clusters is visualized in Fig. 1B. More details on the clusters can be found in Table S5 and a detailed visual overview can be found in Fig. S4.

## Discussion

In this study, we found that 29.3% of the women from the birth cohort Generation R had an unplanned pregnancy. Many factors were associated with unplanned pregnancies. In women, the most important predictors were a young age, migration background, lower educational level, lower household income, financial difficulties, being single, lower cognitive ability, drug use prior to pregnancy, multiple sexual partners in the year prior to the pregnancy, age of first sexual contact < 16 years, and a history of previous abortion. In men, the most important predictors were young age, migration background, lower educational level, drug use prior to pregnancy, and multiple sexual partners in the year prior to pregnancy.

Women with prolonged ambivalent feelings about the unplanned pregnancy were different in terms of several factors; they were more often aged over 35 years, married and already had ≥3 children, and also were older at first sexual contact. These women also more frequently had a Turkish or Moroccan background, Islamic religion, no job, mental health problems (history of depression / anxiety) and reported childhood adversities. Interestingly, these women also relatively often had a high educational level.

In addition, the cluster analyses identified four clusters of women and four clusters of men with unplanned pregnancy. Some of these clusters were in line with findings from the univariate logistic regression analyses, such as clusters 3 and 4 in women and clusters 1 and 4 in men (participants with younger age, lower SEP, single, more mental health problems and/or childhood trauma, substance use and more sexual partners in the last year). The other clusters were more surprising, because only few of the predictors identified in the univariate logistic regression analyses were present in these clusters. This indicates that characteristics of women and men with unplanned pregnancies are very heterogeneous.

Strengths of this study were the large sample size and the availability of multitude of predictors to study in both women and men. Also, the use of cluster analyses to study the co-occurrence of characteristics resulted in additional information as compared to current existing literature on single risk factors of unplanned pregnancy. For example, the univariate logistic regression analyses showed that women with less alcohol intake prior to pregnancy more often had an unplanned pregnancy (Table 1). This contradicts previous studies showing that those involved in binge drinking and substance use more often had an unplanned pregnancy [21, 23]. By clustering data points with close proximity together in a cluster, we identified that women from cluster two relatively often had a Moroccan or Turkish and Islamic background, while women from cluster three more often had a Cape Verdean, Dutch Antilles or Surinam and Christian background. Both clusters had less alcohol intake as compared to the other, mainly non-religious clusters. This suggests that less alcohol intake may be more or less an indication of cultural beliefs and religion rather than an independent protective factor for unplanned pregnancy. Thus, the cluster analysis gives us a more comprehensive understanding of how predictors should be interpreted. Among the limitations is the cross-sectional design of the study, which hampered us to infer causality. Another limitation is that information on pregnancy planning was only obtained from women. Research showed that pregnancy intention is often similar for couples, but this may have influenced our findings [45]. Furthermore, the Generation R Study is a cohort for ongoing pregnancy, hence women who were considering an induced abortion did not participate. Women who continued an unwanted pregnancy may also less likely have participated which may have influenced our findings. Men participated in the Generation R study in 75.1% of the planned pregnancies and 56.6% of the unplanned pregnancies. Since non-participating partners relatively often had a migration background, were younger, and exhibited more risk behaviors (sexual partners, smoking and drug use), this is likely to have diluted some of our findings. Moreover, of the women who participate in Generation R with multiple pregnancies (*n* = 642), we only included the first pregnancy of women. Among these women, the first pregnancy was less often unplanned (19.5%) than the second or third pregnancy (25.5%). Although this difference is small, our choice to study the first pregnancy may have influenced our findings. The number of unplanned pregnancies might have been reduced currently Finally, we were limited by the categorical nature of our assessment of pregnancy planning. Pregnancy intention is complex to measure and could perhaps be better captured in a scale, such as the London Measure of Unplanned Pregnancy [46, 47].

The first cluster of women with an unplanned pregnancy (*n* = 721) consisted of relatively older women with a higher socioeconomic position and higher cognitive ability as compared to the other clusters. They most often had a Dutch background and were not religious. Almost all of them had a partner, had more than one close friend, and an average level of relational difficulties, suggesting that they receive sufficient social support. They on average did not score very high or low on perceived parental rearing and childhood trauma. Still, 40% of them had a history of depression, which is much higher than the group of women with planned pregnancy and also higher than most of the other clusters of women with unplanned pregnancy. As many of the women in this cluster are probably not identified as vulnerable by health care professionals, they may less likely receive extra (mental) health care during and/or after pregnancy. However, some factors that were associated with still ambivalent feelings towards unplanned pregnancy (Table 2) are also seen in the first female cluster. Especially age > 35 years, higher educational level and history of depression were associated with prolonged ambivalent feelings towards the pregnancy. Since parents with ambivalent feelings towards the pregnancy may experience less connection with the fetus and newborn child [48–50], the results of this study suggests that additional support in bonding, attachment and parent-child interaction during and after pregnancy is recommended in this cluster [51].

The second cluster (*n* = 518) consisted mainly of married, multiparous women with a Moroccan or Turkish and often Islamic religion. Previous research showed an increased risk of repeated abortions in Turkish and Moroccan women [52]. A possible explanation could be a taboo about sex education in Islamic families [53, 54], which may be illustrated by the relatively older age at first sexual contact of this cluster. Also, access to care may play a role, as it has been shown that general practitioners less likely discuss and prescribe contraceptives to migrant women in the Netherlands [55]. The women in this cluster perceived high levels of overprotection by their parents, combined with a lower self-esteem and a lower cognitive ability. Possibly, this impacts the health literacy skills resulting in less pro-active family planning. Many characteristics that were associated with prolonged ambivalent feelings towards unplanned pregnancy (Table 2) are also seen in this cluster. In particular there is overlap with Turkish or Moroccan background, Islamic religion, being married, experiencing more financial difficulties, and having multiple children. Hence, these factors cluster both in unplanned pregnancy and prolonged ambivalent feelings towards pregnancy.

The third (*n* = 665) and fourth (*n* = 208) clusters consisted both of relatively young, mainly single women with lower educational level and less financial opportunities. Most striking is perhaps the high percentage of history of induced abortion (50%) in these clusters. Previous research also suggested that history of unplanned pregnancy is associated with an increased risk of subsequent unplanned pregnancy [22, 56, 57]. Non-voluntary first intercourse, sex trade and physical, psychological, or sexual violence or abuse have been identified as risk factors for multiple unintended pregnancies in women and men [57, 58]. In the study of Makenzius et al. (2012), men suggested that increased access to contraception counselling and improved sex and relationship education in school could potentially have prevented the repeated abortion [58]. The use of intrauterine devices was identified to prevent unintended pregnancies in a systematic review about women with multiple unintended pregnancies [57]. Women from the fourth cluster were most often Dutch, while women from the third cluster were more often from Cape Verde, Dutch Antilles and Surinam. These migration backgrounds were strongly associated (OR > 3) with unplanned pregnancy in both women and men. Previous research suggested limited knowledge around and a negative attitude towards contraceptives, probably due to complex socio-cultural factors [59, 60]. However, women from the third cluster also experienced more childhood trauma, more relational difficulties, had a lower self-esteem and lower cognitive ability which may play a role in the circumstances that could result in unplanned pregnancy too. Women in the fourth cluster had even more mental health problems, more childhood trauma and additionally committed more petty or serious crimes, had more substance use and had their first sexual contact at a relatively young age. An association between childhood abuse and unplanned pregnancies was also found by the Norwegian Mother and Child Cohort Study and remained after adjustment for age, migration background and education [61]. Depression and stress symptoms have been associated with less use of contraceptives and unplanned pregnancy [24, 62]. Anxiety, migration background and lower income were all associated with inconsistent use of oral contraceptive use [63]. Lower education seems to be associated with unintended pregnancies independent of contraceptive use and depression rates [64], suggesting that factors such as health literacy and the ability to estimate risks may play a role here. Yet, identifying causality and cascading patterns is extremely difficult, especially because many of them co-occur. Mental health problems are more common in those with lower socioeconomic position, and those with mental health problems may use drugs more frequently [65, 66]. The results of this study suggest that a combination of several predictors together may increase the risk of an unplanned pregnancy within these two clusters of women.

In men, the first (*n* = 168) and fourth (*n* = 133) cluster consisted of men with a lower educational level who experienced more relational difficulties. Similar as in women, other studies also reported lower socioeconomic position as predictor for unplanned pregnancy in men [28, 29]. Men in the first cluster were relatively young, often had a history of serious crimes, more often smoked prior to pregnancy and all of them (occasionally) used drugs prior to pregnancy. In particular marijuana has been identified as a risk factor for the non-use of contraceptives in male and female adolescents, potentially due to inhibited decision making abilities and decreased cognitive function caused by marijuana [67]. Men from the fourth cluster all had multiple sexual partners in the year prior to pregnancy and were more often treated for an STD. Previous research also suggested that having more lifetime sexual partners was associated with unplanned pregnancy in men [28]. In contrast to the clusters of women, migration background did not seem to be a discriminating factor in the clusters of men. Our non-response analyses showed that men with a migration background less likely participated in the study, which may have influenced the cluster analyses.

The second (*n* = 623) and third (*n* = 162) clusters of men seem relatively similar to the first cluster of women. They were relatively older and more often higher educated than those from the first and fourth cluster and they more often had a job (87%) than those from the first cluster. Men in the second cluster experienced little relational difficulties and only few of them (1.1%) had a history of depression. Men in the third cluster were even more often higher educated (47%). In contrast to the second cluster, men in the third cluster had a very high percentage of history of depression (84%) and anxiety (47%). They also reported higher values of relational difficulties and more often had a history of serious crimes. Limited research on mental health as predictor for unplanned pregnancy in men has been conducted. The results from this study suggests that mental health factors do not only play a role in women but are also involved in reproductive choices in men.

The number of unplanned pregnancies in this cohort (29%) was as expected compared to previous studies showing that 36% of all pregnancies in Western Europe were unplanned [1] and that 20% of the women in the Netherlands ever experienced an unintended pregnancy [3]. The rate of unintended pregnancy declined between 1994 and 2001 in the United States. However, it only reduced among adolescents, college students and women from higher socioeconomic position, while it increased in socioeconomic disadvantaged women [68]. International behavior change interventions mostly focused on adolescents or specific groups such as STD clinic patients, people living with human immunodeficiency virus (HIV), men who have sex with men and women working in the sex industry [69]. This shows the importance of developing preventive inclusive interventions, such as incorporating reproductive health promotion into primary care [70]. For example, the identification of the second cluster of mainly married, multiparous Moroccan or Turkish women with an unplanned pregnancy suggests the need for a larger role for the general practitioner to inquire about family planning and the need for contraception in a culturally appropriate manner. Importantly, also women who do not proactively consult the general practitioner themselves should be reached in this respect. Furthermore, clusters three and four of women and clusters one and four of men consisted mainly of relatively young, single individuals with a lower socioeconomic position. Although it is not clear whether their unplanned pregnancy resulted from a financial inability to purchase contraception, testing with no-costs contraception may be worthwhile in this group. Research has shown that providing no-costs contraception could reduce unintended pregnancies in particular among women with a history of induced abortion, which is the case in about half of the women in both clusters three and four in our study [71]. Thus, physicians working in abortion clinics as well family doctors, primary care physicians and/or general practitioners are encouraged to pay more attention to contraception counseling after an induced abortion. Furthermore, the program Nu Niet Zwanger (Not Pregnant Now) was initiated in 2014 in the Netherlands to prevent unintended pregnancy in men and women who were identified as living in vulnerable situations (such as substance abuse, youth protection, homeless care or social work). The aim of the program was to start the conversation about contraception from a strong emphasis of building a relationship of trust and out-reaching care and building bridges between the social and medical domain [72]. This program seems very applicable to some women from cluster four and men from cluster one who experienced relational difficulties and had a history of childhood trauma, serious crimes and substance use. However, our largest clusters (cluster one of women and clusters two and three of men) consisted of women and men with unplanned pregnancy without severe financial difficulties, substance abuse or childhood trauma. These individuals are more likely to be overseen by current programs. Investigating their specific needs regarding contraception counseling should be a focus for future research. Finally, all practitioners involved with couples in the fertile period could start to ask the question: “Would you like to become pregnant in the next year? ” [73]. This allows for preconception care in those who do wish to become pregnant in the next year and contraception counseling in those who do not want to become pregnant (in the next year).

## Conclusions

The results from our study show that many predictors were associated with unplanned pregnancy. Although less data was available for men, the predictors that were available for both sexes showed similar associations, suggesting that similar mechanisms occur. Thus far, unplanned pregnancy in adolescents and women with social adversity received most attention. However, the results from our study show new patterns of vulnerability around reproductive choice: firstly, related to relatively older women with a higher socioeconomic position and higher cognitive ability with often a history of depression; and secondly, related to married, multipara women with a Moroccan or Turkish background. Furthermore, patterns of vulnerability in relation to unplanned pregnancy in men were identified: firstly, men with lower educational level who experience several relational difficulties with additionally a history of serious crimes, smoking and (occasional) substance use or with additionally a pattern of multiple sexual partners in the year prior to pregnancy and treatment for STD; and secondly, remarkably, men with higher education and having a job formed a cluster, as well as those with higher prevalence of mental health disorders in relation to unplanned pregnancy. These novel patterns ask for a multi-layered approach in both public health and clinical practice. Clearly a one size fits all intervention seems unlikely to be sufficient, and our results call for heterogeneous measures to prevent unplanned pregnancies or to mitigate the implications of unplanned pregnancies.

## Supplementary Information

**Additional file 1: Figure S1.** Flowchart of the study participants. **Table S1.** Information about the characteristics included in the study and whether information was available for women and men. **Figure S2.** A + B: Visualization of the clusters in a heat map for (A) women and (B) men separately. **Table S2.** Cluster validation parameters women. **Table S3.** Descriptive information of the clusters of women with an unplanned pregnancy. **Table S4.** Cluster validation parameters men. **Table S5.** Descriptive information of the clusters of men with an unplanned pregnancy. **Figure S3.** Boxplots and histograms of all variables stratified per cluster for women. **Figure S4.** Boxplots and histograms of all variables stratified per cluster for men.

## Data Availability

The data underlying this article cannot be shared publicly because participants of the Generation R study were assured raw data would remain confidential and would not be shared to the public. The data underlying the results presented in the study are available on request with a formal data sharing agreement for researchers who meet the criteria for access to confidential data. Requests should be directed toward the management team of the Generation R study (secretariaat.genr@erasmusmc.nl).

## Abbreviations

UP: Unplanned pregnancy
STD: Sexually transmitted disease
