# Supplementary material for: Clustering of characteristics associated with unplanned pregnancies: the generation R study
Source: BMC Public Health. 2022 Oct 24;22:1957. doi: 10.1186/s12889-022-14342-y (PMC9590126; doi:10.1186/s12889-022-14342-y)
Supplement: Supplementary file 1 — Additional file 1: Figure S1. Flowchart of the study participants. Table S1. Information about the characteristics included in the study and whether information was available for women and men. Figure S2. A + B: Visualization of the clusters in a heat map for (A) women and (B) men separately. Table S2. Cluster validation parameters women. Table S3. Descriptive information of the clusters of women with an unplanned pregnancy. Table S4. Cluster validation parameters men. Table S5. Descriptive information of the clusters of men with an unplanned pregnancy. Figure S3. Boxplots and histograms of all variables stratified per cluster for women. Figure S4. Boxplots and histograms of all variables stratified per cluster for men. [file 12889_2022_14342_MOESM1_ESM.docx]

**Clustering of characteristics associated with unplanned pregnancies: The Generation R Study**

**Supplemental material**

Table of content

[Figure S1: Flowchart of the study participants. 2](#_Toc115163705)

[Table S1: Information about the characteristics included in the study and whether information was available for women and men. 3](#_Toc115163706)

[Figure S2 A + B: Visualization of the clusters in a heat map for (A) women and (B) men separately. 6](#_Toc115163707)

[Table S2: Cluster validation parameters women 7](#_Toc115163708)

[Table S3: Descriptive information of the clusters of women with an unplanned pregnancy 8](#_Toc115163709)

[Table S4: Cluster validation parameters men 10](#_Toc115163710)

[Table S5: Descriptive information of the clusters of men with an unplanned pregnancy 11](#_Toc115163711)

[Figure S3: Boxplots and histograms of all variables stratified per cluster for women. 12](#_Toc115163712)

[Figure S4: Boxplots and histograms of all variables stratified per cluster for men. 13](#_Toc115163713)

[References 14](#_Toc115163714)

**
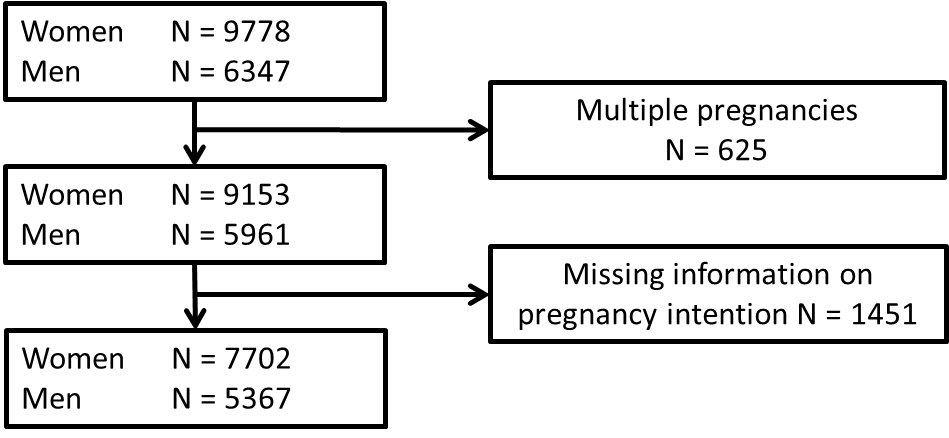
**

# Figure S1: Flowchart of the study participants.

Table S1: Information about the characteristics included in the study and whether information was available for women and men. **All information was obtained by questionnaire unless indicated otherwise.**

|  | Women | Men | Information |
| --- | --- | --- | --- |
| *Demographic information* | | | |
| Age at conception | X | X | Age at birth child minus the gestational age of the child at birth, categorized into less than 20 years, 20 to 25 years, 25 to 30 years, 30 to 35 years and 35 years or older. |
| Ethnic background | X | X | Ethnic background was defined according the classification of Statistics Netherlands: (1) if both parents are born in the Netherlands, the ethnic background is Dutch; (2) if one of the parents is born in another country than the Netherlands, that country counts; (3) if both parents are born in the same country other than the Netherlands, that country counts; (4) if the parents are born in the different countries other than the Netherlands, the country of mothers counts; and (5) if that person and both parents are born in different countries other than the Netherlands, the country of birth of that specific person counts (1). Categorized into Dutch, Indonesian, Cape Verdean, Moroccan, Dutch Antilles, Surinamese, Turkish, European, Asian and Other (African, American and Oceanian). |
| Religion | X |  | Christian, Hindustan, Islamic, Other religions and No religion. |
| Education | X | X | Highest attained educational level, categorized as:  Low (primary school; lower vocational training; intermediate general school; 3 years general secondary school), which typically corresponds to ≤12 years of education;  Mid‐low (>3 years general secondary school; intermediate vocational training; 1st year higher vocational training), in general corresponding with 13–15 years of education;  Mid‐high (higher vocational training; Bachelor's degree), typically matching with 16 or 17 years of education;  And High (higher academic education; PhD), usually indicating 18 years of education or more. |
| Paid job | X | X | Having a paid job (including being self-employed) versus Having no paid job. |
| Net household income | X |  | Categorized as: Less than €1200/month (social security level); Between €1200 and 2000/month; and More than €2000/month (modal income). |
| Financial difficulties | X |  | Difficulty in paying food, rent, electricity bill and suchlike, categorized into: No difficulty; Some difficulty; and Great difficulty. |
| Marital status | X |  | Categorized as: Being married, Cohabiting; and Single. |
| Parity | X |  | Based on medical records, categorized as: Nulliparous, one child, two children or ≥ three children. |
| *Mental health* | | | |
| Childhood Trauma | X |  | The 34-item short version of the Childhood Trauma Questionnaire (CTQ) with an internal consistency of 0.92 (2). Items assess physical neglect, emotional neglect, physical abuse, emotional abuse and sexual abuse which together form an Overall Trauma Score. The items were transformed into a z-score with mean zero and standard deviation of one. |
| Perceived parental rearing | X |  | A validated 23-item short form of the ‘Own memories on parenting questionnaire’ (s-EMBU) (3, 4). The internal consistency was 0.87 and 0.85 for emotional warmth, 0.88 and 0.80 for rejection and 0.61 and 0.61 for overprotection for the women’s father and mother, respectively. The average values of emotional warmth, rejection and overprotection were calculated for the parents together. The items were transformed into a z-score with mean zero and standard deviation of one. |
| History of depression | X | X | Vignette explaining what depression is followed by questions about whether the individual ever had these symptoms and received treatment for it. Self-reported history of depression was compared in a subsample of the cohort (n=928 women and n=827 men) with the Composite International Diagnostic Interview (CIDI) resulting in a sensitivity of 77% and 76% a specificity of 80% and 87% for respectively women and men (5). |
| History of anxiety | X | X | Similarly to depression, a vignette assessed the history of anxiety. Self-reported history of anxiety had a sensitivity of 40% and 27% and a specificity of 91% and 93% for respectively women and men compared with the CIDI (5). |
| History of eating disorder | X |  | Similarly to depression, a vignette about history of anorexia or bulimia nervosa. Self-reported lifetime eating disorder had a sensitivity of 95% and a specificity of 94% as compared to the diagnosis of lifetime depression obtained with the CIDI (5). |
| Self-esteem | X |  | The Rosenberg Self-Esteem Scale, a 10-item questionnaire with an internal consistency of 0.79 (6), assessing items such as “I feel that I have a number of good qualities” and “At times I feel that I am no good at all”. The items were transformed into a z-score with mean zero and standard deviation of one. |
| Cognitive ability | X |  | A 12-item reliable and validated short version of the Raven's Progressive Matrices (7). Categorized into <70 (mild intellectual disability), 70-85 (borderline intellectual functioning) and ≥85 (normal intellectual functioning) (8). |
| *Physical health* |  |  |  |
| Chronic somatic disease | X | X | The participants were considered to have a chronic somatic disease if they reported to be under treatment by a family doctor or specialist in the last year for one or more of the following diseases: diabetes, raised cholesterol level, high blood pressure, heart condition, chronic eczema, severe intestinal disorder, systematic lupus erythematosus (only in women), arthritis, multiple sclerosis thyroid disorder or chronic bronchitis. |
| BMI prior to pregnancy | X | X | Height (cm) and weight (kg) were measured without shoes and heavy clothing. Information about the women’s weight just before pregnancy was obtained by questionnaire. BMI (kg/m2) was calculated and categorized into underweight (<20 kg/m2), normal weight (20-24.9 kg/m2), overweight (25-29.9 kg/m2), and obese (≥30 kg/m2). |
| *Social factors* | | | |
| Relational difficulties | X | X | The Dutch long-lasting difficulties (LLD) list containing of 16 items, which addresses problem situations in the preceding year. Women reported whether they have had difficulties with family members, friends, people from the neighborhood, difficulties at school/work, and reported whether sexual, financial or housing problems had occurred. The internal consistency of LLD in this sample was 0.72 for the women and 0.63 for the men (9). |
| History of delinquency | X | X | 18 items regarding different types of delinquent behavior (10), categorized into: No crimes; Petty crimes; and Serious crimes. |
| Number of good friends | X |  | Categorized into ≤1 good friend or >1 good friend. |
| *Substance use* | | | |
| Alcohol use | X | X | Alcohol use before pregnancy, categorized into: <1 glass per week; 1-6 glasses per week; and ≥1 glasses per day. This was reported by the women about themselves and about their partners. |
| Smoking | X | X | Smoking before pregnancy, categorized into: No; <5 cigarettes per day; and ≥5 cigarettes per day. This was reported by the women about themselves and about their partners. |
| Drug use | X | X | Drug use before pregnancy (i.e. marihuana, hashish, cocaine, heroin, or ecstasy) categorized into: Yes; and No. This was reported by the women about themselves and about their partners. |
| *Sexual behavior* | | | |
| Number of sexual partners | X | X | Having had more than one sexual partner in the year prior to pregnancy. This was reported by the women about themselves and about their partners. |
| STD treatment | X | X | Whether or not the participant had ever been treated for a sexually transmitted disease. This was reported by the women about themselves and about their partners. |
| Age of first sexual contact | X |  | Age of first sexual contact, categorized into: <16 years; 16-19 years; and ≥20 years. |
| Prior induced abortion | X |  | Whether or not the woman ever had an induced abortion, categorized into: Yes; and No. |

X indicates whether the information was available for women and/or men.


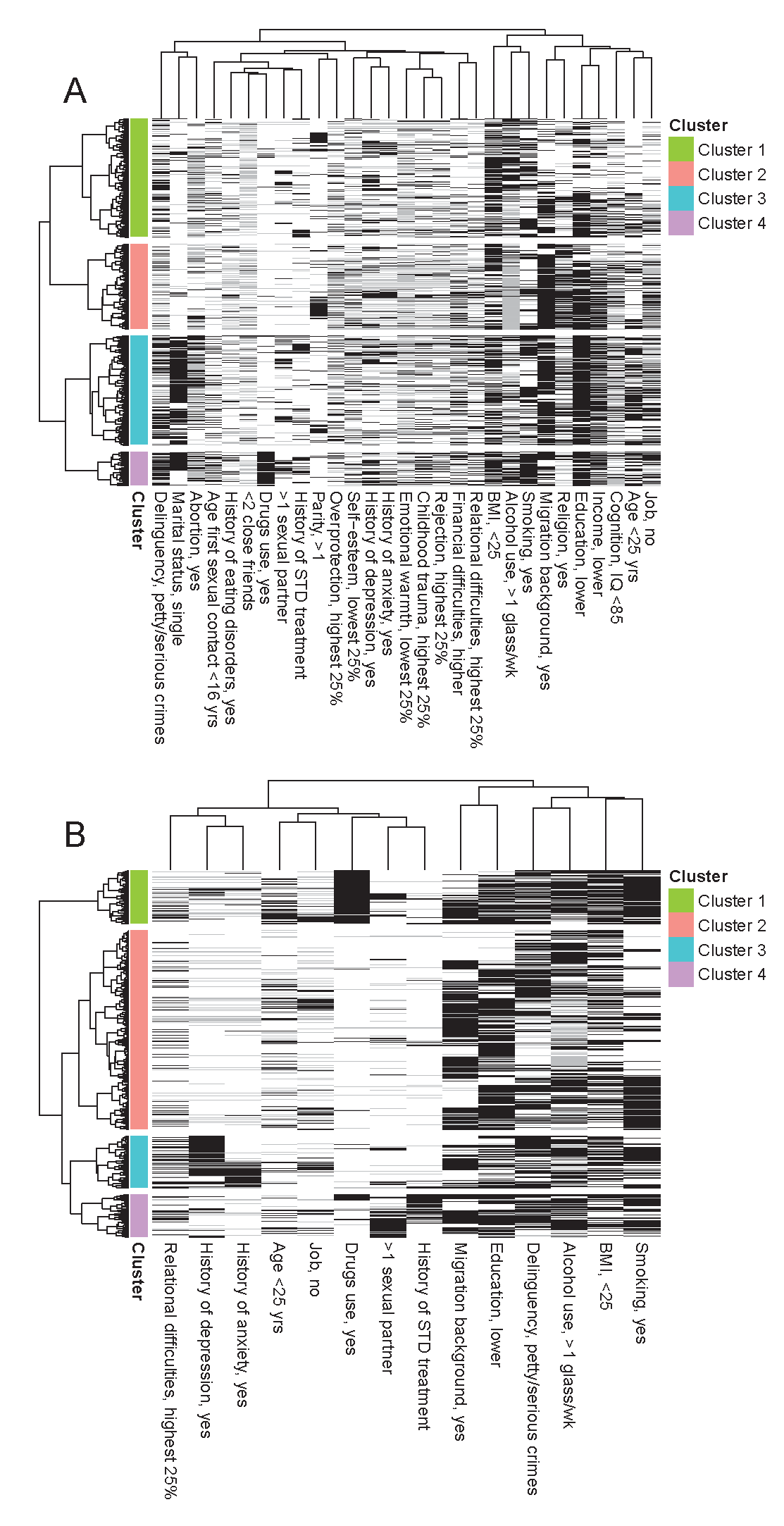


Figure S2 A + B: Visualization of the clusters in a heat map for (A) women and (B) men separately.
The columns represent the characteristics and each horizontal line represents one person. The four clusters are depicted with a blank space between them and illustrated by the dendrogram on the left side of the image. The dendrogram on the top shows the proximity of the different characteristics. All characteristics were dichotomized; the categories as mentioned in the variable names are depicted in black color in the heatmap. The white color depicts the opposite category and missing values were depicted in grey color.

# Table S2: Cluster validation parameters women

| **Number of clusters** | **2** | **3** | **4** | **5** | **6** | **7** | **8** |
| --- | --- | --- | --- | --- | --- | --- | --- |
| **Average distance between** | 0.40 | 0.39 | 0.39 | 0.39 | 0.38 | 0.38 | 0.38 |
| **Average distance within** | 0.34 | 0.32 | 0.31 | 0.30 | 0.30 | 0.29 | 0.29 |
| **Average Silhouette width** | 0.13 | 0.12 | 0.13 | 0.10 | 0.08 | 0.09 | 0.08 |
| **Dunn index** | 1.11 | 1.03 | 1.11 | 1.01 | 0.95 | 0.89 | 0.87 |
| **Separation index** | 0.03 | 0.03 | 0.02 | 0.02 | 0.02 | 0.02 | 0.02 |

# Table S3: Descriptive information of the clusters of women with an unplanned pregnancy

| **Cluster** | **Cluster 1 (N=721)** | **Cluster 2 (N=518)** | **Cluster 3 (N=665)** | **Cluster 4 (N=208)** |
| --- | --- | --- | --- | --- |
| **Age; years (SD)** | 29.65 (5.43) | 28.45 (5.19) | 25.16 (5.98) | 24.63 (5.41) |
| **Ethnic background** |  |  |  |  |
| Dutch | 441 (61.5%) | 47 (9.2%) | 111 (16.9%) | 85 (41.3%) |
| Indonesian | 27 (3.8%) | 6 (1.2%) | 15 (2.3%) | 10 (4.9%) |
| Cape Verdian | 38 (5.3%) | 12 (2.3%) | 96 (14.6%) | 25 (12.1%) |
| Moroccan | 5 (0.7%) | 115 (22.5%) | 10 (1.5%) | 4 (1.9%) |
| Dutch Antilles | 38 (5.3%) | 9 (1.8%) | 101 (15.4%) | 12 (5.8%) |
| Surinamese | 54 (7.5%) | 16 (3.1%) | 216 (32.9%) | 35 (17.0%) |
| Turkish | 10 (1.4%) | 192 (37.6%) | 14 (2.1%) | 5 (2.4%) |
| European | 49 (6.8%) | 45 (8.8%) | 26 (4.0%) | 19 (9.2%) |
| Asian | 19 (2.6%) | 38 (7.4%) | 11 (1.7%) | 2 (1.0%) |
| Other | 36 (5.0%) | 31 (6.1%) | 56 (8.5%) | 9 (4.4%) |
| **Educational level** |  |  |  |  |
| Low | 153 (21.6%) | 203 (41.9%) | 349 (54.3%) | 102 (50.0%) |
| Mid-low | 247 (34.9%) | 170 (35.1%) | 253 (39.3%) | 76 (37.3%) |
| Mid-high | 169 (23.9%) | 58 (12.0%) | 33 (5.1%) | 17 (8.3%) |
| High | 139 (19.6%) | 53 (11.0%) | 8 (1.2%) | 9 (4.4%) |
| **Paid job, yes** | 456 (78.4%) | 135 (42.7%) | 161 (39.8%) | 46 (36.5%) |
| **Household income** |  |  |  |  |
| <€1200/month | 71 (12.4%) | 128 (38.9%) | 302 (72.4%) | 87 (63.0%) |
| €1200-2000/month | 134 (23.4%) | 102 (31.0%) | 89 (21.3%) | 30 (21.7%) |
| ≥€2000/month | 368 (64.2%) | 99 (30.1%) | 26 (6.2%) | 21 (15.2%) |
| **Financial difficulties** |  |  |  |  |
| No | 460 (79.0%) | 193 (60.5%) | 201 (48.7%) | 57 (43.8%) |
| Some | 112 (19.2%) | 92 (28.8%) | 157 (38.0%) | 55 (42.3%) |
| Great | 10 (1.7%) | 34 (10.7%) | 55 (13.3%) | 18 (13.8%) |
| **Religion** |  |  |  |  |
| Not religious | 452 (76.1%) | 44 (13.5%) | 245 (55.4%) | 109 (77.3%) |
| Christian | 97 (16.3%) | 69 (21.1%) | 116 (26.2%) | 23 (16.3%) |
| Hindustan | 13 (2.2%) | 2 (0.6%) | 28 (6.3%) | 1 (0.7%) |
| Islamic | 19 (3.2%) | 194 (59.3%) | 34 (7.7%) | 4 (2.8%) |
| Other religion | 13 (2.2%) | 18 (5.5%) | 19 (4.3%) | 4 (2.8%) |
| **Marital status** |  |  |  |  |
| Married | 151 (21.3%) | 460 (92.2%) | 45 (6.9%) | 8 (3.9%) |
| Cohabiting | 502 (70.9%) | 26 (5.2%) | 71 (11.0%) | 81 (39.9%) |
| Single | 55 (7.8%) | 13 (2.6%) | 532 (82.1%) | 114 (56.2%) |
| **Parity** |  |  |  |  |
| 0 | 465 (64.9%) | 214 (43.1%) | 414 (63.4%) | 169 (81.2%) |
| 1 | 150 (20.9%) | 140 (28.2%) | 152 (23.3%) | 31 (14.9%) |
| 2 | 91 (12.7%) | 83 (16.7%) | 72 (11.0%) | 8 (3.8%) |
| ≥3 | 11 (1.5%) | 59 (11.9%) | 15 (2.3%) | 0 (0.0%) |
| **Childhood trauma score (SD)** | 0.09 (1.01) | 0.06 (0.94) | 0.43 (1.28) | 1.00 (1.63) |
| **Perceived parental rearing** |  |  |  |  |
| Emotional warmth, score (SD) | -0.01 (1.12) | -0.05 (0.97) | -0.18 (0.61) | -0.10 (1.44) |
| Overprotection, score (SD) | -0.04 (0.99) | 0.24 (1.06) | 0.27 (1.07) | 0.13 (1.12) |
| Rejection, score (SD) | 0.06 (1.05) | 0.06 (0.93) | 0.37 (1.35) | 0.67 (1.52) |
| **History of depression; yes** | 235 (40.4%) | 106 (31.3%) | 137 (29.0%) | 86 (55.8%) |
| **History of anxiety; yes** | 109 (18.6%) | 73 (20.9%) | 81 (16.7%) | 45 (29.4%) |
| **History of eating disorder; yes** | 72 (12.6%) | 37 (11.5%) | 35 (7.4%) | 31 (20.8%) |
| **Self-esteem; score (SD)** | 0.01 (0.99) | -0.37 (1.13) | -0.41 (1.19) | -0.38 (1.24) |
| **Cognition; IQ (SD)** | 97.09 (13.89) | 88.41 (16.18) | 87.85 (15.13) | 92.39 (12.19) |
| **BMI prior to pregnancy (SD)** | 23.46 (4.66) | 24.33 (4.73) | 23.81 (5.07) | 22.44 (4.24) |
| **Relational difficulties; score (SD)** | 0.08 (1.01) | 0.25 (1.17) | 0.68 (1.28) | 1.00 (1.29) |
| **History of delinquency** |  |  |  |  |
| No crimes | 326 (55.8%) | 262 (75.3%) | 187 (38.6%) | 21 (13.6%) |
| Petty crimes | 103 (17.6%) | 56 (16.1%) | 128 (26.4%) | 34 (22.1%) |
| Serious crimes | 155 (26.5%) | 30 (8.6%) | 170 (35.1%) | 99 (64.3%) |
| **≤1 Close friend; yes** | 14 (3.6%) | 25 (9.0%) | 50 (12.7%) | 13 (11.0%) |
| **Alcohol use prior to pregnancy** |  |  |  |  |
| <1 glass/week | 202 (38.6%) | 69 (55.6%) | 272 (67.2%) | 63 (37.1%) |
| 1-6 glasses/week | 254 (48.6%) | 44 (35.5%) | 118 (29.1%) | 76 (44.7%) |
| ≥1 glass/day | 67 (12.8%) | 11 (8.9%) | 15 (3.7%) | 31 (18.2%) |
| **Smoking prior to pregnancy** |  |  |  |  |
| No | 338 (50.8%) | 350 (72.8%) | 341 (54.0%) | 15 (7.8%) |
| <5 cigarettes/day | 92 (13.8%) | 44 (9.1%) | 95 (15.0%) | 41 (21.2%) |
| ≥5 cigarettes/day | 235 (35.3%) | 87 (18.1%) | 196 (31.0%) | 137 (71.0%) |
| **Drug use prior to pregnancy; yes** | 37 (5.3%) | 4 (0.8%) | 29 (4.5%) | 192 (95.5%) |
| **>1 sexual partner; yes** | 87 (13.0%) | 5 (1.0%) | 128 (21.1%) | 52 (27.5%) |
| **History of treatment for STD; yes** | 102 (15.4%) | 10 (2.1%) | 129 (21.2%) | 60 (31.2%) |
| **Age of first time sexual contact; years (SD)** | 17.39 (2.71) | 19.92 (3.34) | 17.16 (2.50) | 15.62 (1.63) |
| **History of induced abortion; yes** | 127 (32.4%) | 63 (17.7%) | 206 (50.2%) | 51 (49.5%) |

# Table S4: Cluster validation parameters men

| **Number of clusters** | **2** | **3** | **4** | **5** | **6** | **7** | **8** |
| --- | --- | --- | --- | --- | --- | --- | --- |
| **Average distance between** | 0.38 | 0.36 | 0.37 | 0.35 | 0.35 | 0.34 | 0.33 |
| **Average distance within** | 0.29 | 0.27 | 0.26 | 0.25 | 0.24 | 0.23 | 0.23 |
| **Average Silhouette width** | 0.24 | 0.19 | 0.19 | 0.12 | 0.13 | 0.09 | 0.08 |
| **Dunn index** | 1.30 | 0.96 | 0.92 | 0.74 | 0.85 | 0.75 | 0.73 |
| **Separation index** | 0.03 | 0.01 | 0.01 | 0.00 | 0.00 | 0.00 | 0.00 |

# Table S5: Descriptive information of the clusters of men with an unplanned pregnancy

| **Cluster** | **Cluster 1 (N=168)** | **Cluster 2 (N=623)** | **Cluster 3 (N=162)** | **Cluster 4 (N=133)** |
| --- | --- | --- | --- | --- |
| **Age; years (SD)** | 27.44 (6.85) | 31.19 (6.60) | 33.13 (6.40) | 30.21 (6.76) |
| **Migration background** |  |  |  |  |
| Dutch | 79 (49.4%) | 249 (44.1%) | 90 (58.8%) | 57 (43.8%) |
| Indonesian | 5 (3.1%) | 21 (3.7%) | 8 (5.2%) | 8 (6.2%) |
| Cape Verdian | 11 (6.9%) | 30 (5.3%) | 2 (1.3%) | 11 (8.5%) |
| Moroccan | 0 (0.0%) | 30 (5.3%) | 8 (5.2%) | 4 (3.1%) |
| Dutch Antilles | 17 (10.6%) | 17 (3.0%) | 7 (4.6%) | 19 (14.6%) |
| Surinamese | 25 (15.6%) | 65 (11.5%) | 8 (5.2%) | 19 (14.6%) |
| Turkish | 6 (3.8%) | 56 (9.9%) | 17 (11.1%) | 7 (5.4%) |
| European | 11 (6.9%) | 45 (8.0%) | 5 (3.3%) | 1 (0.8%) |
| Asian | 3 (1.9%) | 22 (3.9%) | 4 (2.6%) | 1 (0.8%) |
| Other | 3 (1.9%) | 30 (5.3%) | 4 (2.6%) | 3 (2.3%) |
| **Educational level** |  |  |  |  |
| Low | 70 (49.3%) | 180 (32.5%) | 25 (16.9%) | 45 (39.1%) |
| Mid-low | 47 (33.1%) | 177 (32.0%) | 53 (35.8%) | 44 (38.3%) |
| Mid-high | 16 (11.3%) | 85 (15.4%) | 34 (23.0%) | 11 (9.6%) |
| High | 9 (6.3%) | 111 (20.1%) | 36 (24.3%) | 15 (13.0%) |
| **Paid job, yes** | 103 (79.8%) | 442 (86.5%) | 123 (87.9%) | 90 (85.7%) |
| **History of depression; yes** | 25 (18.5%) | 6 (1.1%) | 122 (83.6%) | 24 (21.6%) |
| **History of anxiety; yes** | 12 (8.6%) | 7 (1.3%) | 68 (46.9%) | 5 (4.5%) |
| **BMI prior to pregnancy (SD)** | 23.35 (2.84) | 25.50 (3.79) | 24.72 (3.30) | 25.16 (3.24) |
| **Relational difficulties; score (SD)** | 0.54 (1.45) | 0.03 (0.99) | 0.83 (1.33) | 0.57 (1.43) |
| **History of delinquency** |  |  |  |  |
| No crimes | 19 (14.6%) | 234 (47.4%) | 27 (19.1%) | 23 (23.0%) |
| Petty crimes | 14 (10.8%) | 58 (11.7%) | 15 (10.6%) | 9 (9.0%) |
| Serious crimes | 97 (74.6%) | 202 (40.9%) | 99 (70.2%) | 68 (68.0%) |
| **Alcohol use prior to pregnancy** |  |  |  |  |
| <1 glass/week | 40 (27.6%) | 138 (32.2%) | 26 (20.2%) | 26 (23.4%) |
| 1-6 glasses/week | 67 (46.2%) | 188 (43.8%) | 72 (55.8%) | 54 (48.6%) |
| ≥1 glass/day | 38 (26.2%) | 103 (24.0%) | 31 (24.0%) | 31 (27.9%) |
| **Smoking prior to pregnancy** |  |  |  |  |
| No | 17 (10.4%) | 359 (59.8%) | 78 (49.1%) | 46 (35.4%) |
| <5 cigarettes/day | 34 (20.9%) | 96 (16.0%) | 20 (12.6%) | 25 (19.2%) |
| ≥5 cigarettes/day | 112 (68.7%) | 145 (24.2%) | 61 (38.4%) | 59 (45.4%) |
| **Drug use prior to pregnancy; yes** | 162 (99.4%) | 10 (1.7%) | 5 (3.2%) | 28 (21.7%) |
| **>1 sexual partner; yes** | 27 (17.6%) | 17 (3.0%) | 16 (10.7%) | 70 (61.9%) |
| **History of treatment for STD; yes** | 7 (4.6%) | 15 (2.6%) | 8 (5.3%) | 70 (57.4%) |


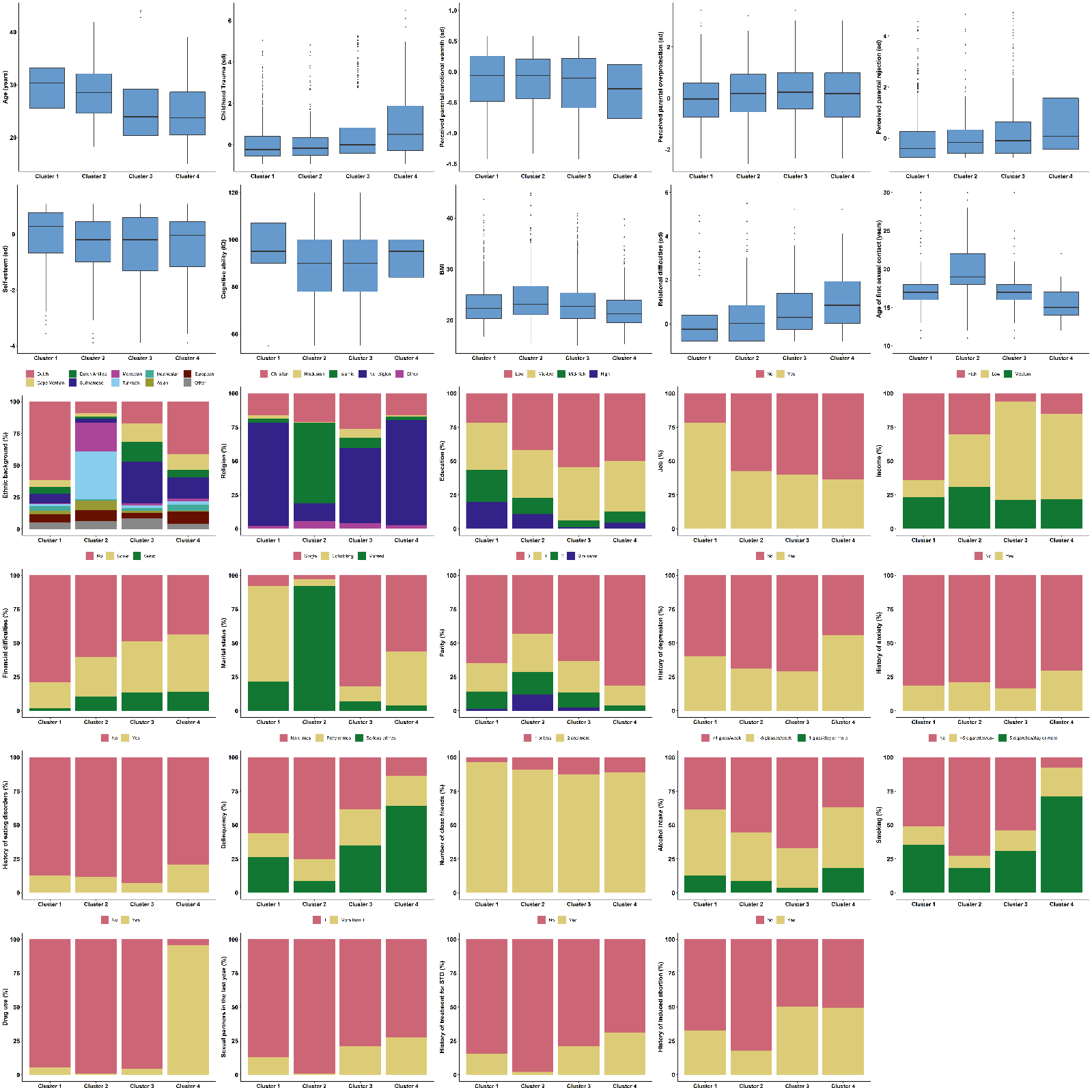


# Figure S3: Boxplots and histograms of all variables stratified per cluster for women.

For the boxplots, the lower and upper hinges represents the interquartile range (the 25th and 75th percentiles), the horizontal line is drawn at the median and the whiskers indicate -1.5*25th percentile and -1.5*75th percentile. Data points beyond the end of the whiskers are plotted individually with dots. The stacked histograms show the percentages of the variable levels for all clusters separately. BMI: body mass index


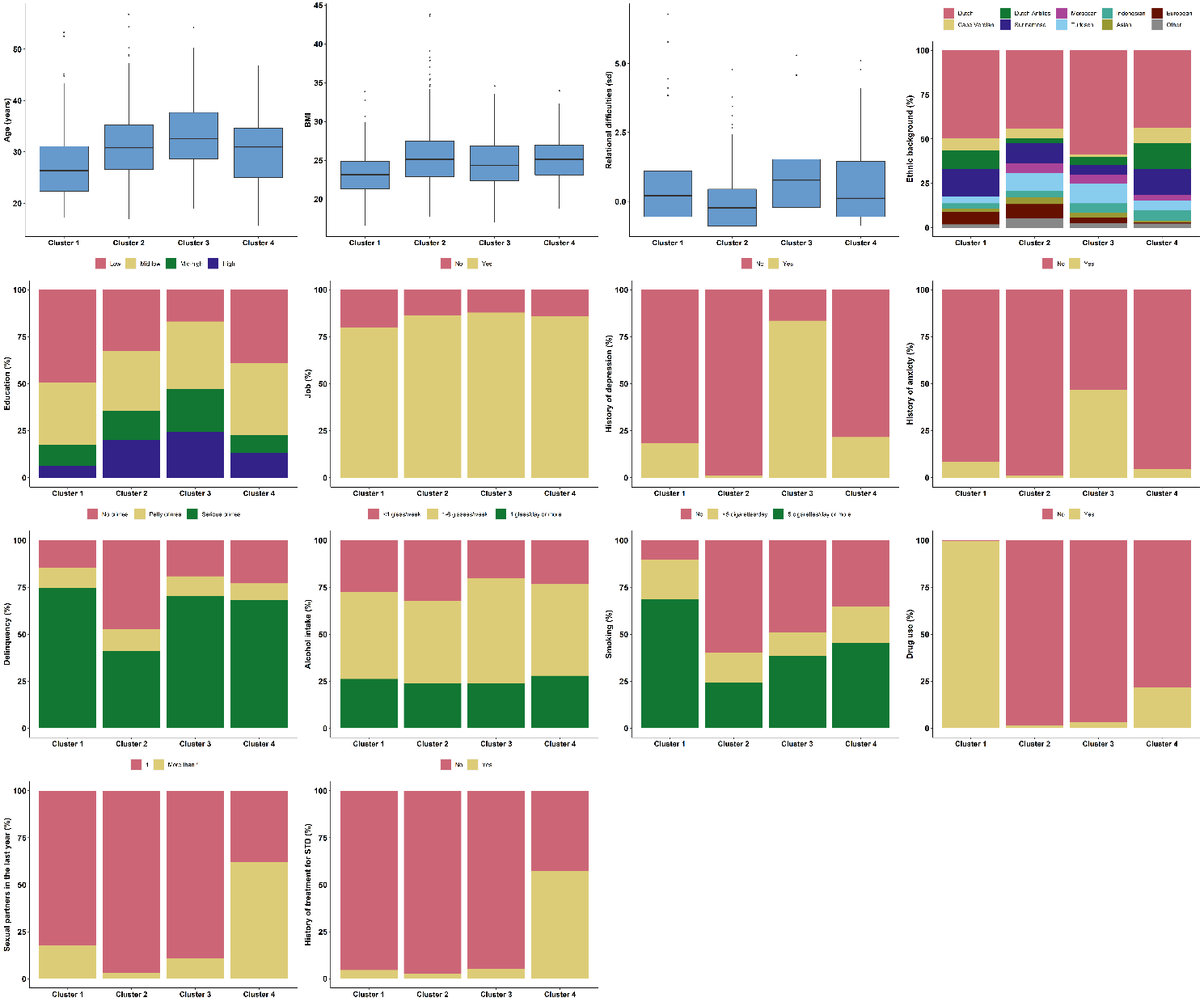


# Figure S4: Boxplots and histograms of all variables stratified per cluster for men.

For the boxplots, the lower and upper hinges represents the interquartile range (the 25th and 75th percentiles), the horizontal line is drawn at the median and the whiskers indicate -1.5*25th percentile and -1.5*75th percentile. Data points beyond the end of the whiskers are plotted individually with dots. The stacked histograms show the percentages of the variable levels for all clusters separately. BMI: body mass index.

# References

1. Jaddoe VWV, Mackenbach JP, Moll HA, Steegers EAP, Tiemeier H, Verhulst FC, et al. The Generation R Study: design and cohort profile. European journal of epidemiology. 2006;21(6):475.

2. Bernstein DP, Fink L, Handelsman L, Foote J, Lovejoy M, Wenzel K, et al. Initial reliability and validity of a new retrospective measure of child abuse and neglect. The American Journal of Psychiatry. 1994;151(8):1132-6.

3. Arrindell W, Richter J, Eisemann M, Gärling T, Rydén O, Hansson S, et al. The short-EMBU* in East-Germany and Sweden: A cross-national factorial validity extension. Scandinavian Journal of Psychology. 2001;42(2):157-60.

4. Perris C, Jacobsson L, Linndström H, von Knorring L, Perris H. Development of a new inventory for assessing memories of parental rearing behaviour. Acta Psychiatrica Scandinavica. 1980;61(4):265-74.

5. Andrews G, Peters L. The psychometric properties of the Composite International Diagnostic Interview. Soc Psychiatry Psychiatr Epidemiol. 1998;33(2):80-8.

6. Rosenberg M. Rosenberg self-esteem scale (RSE). Acceptance and commitment therapy Measures package. 1965;61(52):18.

7. Irrgang M, Dorenkamp M, Reohr P, Vik P. C-28 Raven’s Progressive Matrices: Validation of a Short Form. Archives of Clinical Neuropsychology. 2019;34(6):1057-.

8. Nouwens PJG, Lucas R, Smulders NBM, Embregts PJCM, van Nieuwenhuizen C. Identifying classes of persons with mild intellectual disability or borderline intellectual functioning: a latent class analysis. BMC Psychiatry. 2017;17(1):257.

9. Hendriks AAJ, Ormel J, Van de Willige G. Long lasting difficulties measured with a self-assessment questionnaire and semi structured interview: a theoretical and empirical comparison. Gedrag en Gezondheid. 1990;18:273-83.

10. Van der Laan AM, Blom M, Verwers C, Essers AAM. Jeugddelinquentie: Boom Juridische uitgevers; 2006.
